# Predicting acute kidney injury in type B aortic dissection: development and validation of a clinical nomogram

**DOI:** 10.1016/j.clinsp.2026.101209

**Published:** 2026-10-13

**Authors:** Weijian Chen, Zhengfei Li, Xiaojun Shu

**Affiliations:** Department of Vascular surgery, General Surgery, The First Hospital of Lanzhou University, Gansu, China

**Keywords:** Prediction model, Acute kidney injury, Aortic dissection, Nomogram

## Abstract

**Objectives:**

Acute Kidney Injury (AKI) is a frequent and serious complication in patients with Type B Aortic Dissection (TBAD). Existing prediction models are mainly derived from type A or mixed cohorts and do not account for the distinct pathophysiology of TBAD. This study aimed to develop and validate a nomogram for individualized AKI risk prediction in TBAD patients.

**Methods:**

Data were obtained from the eICU Collaborative Research Database (eICU-CRD) and the Medical Information Mart for Intensive Care IV (MIMIC-IV). The eICU-CRD cohort was used for model development and internal validation, while MIMIC-IV served for external validation. A three-step variable selection strategy combining Least Absolute Shrinkage and Selection Operator (LASSO), univariate, and multivariate logistic regression analyses was applied. Model performance was evaluated using the Area Under the receiver operating characteristic Curve (AUC), calibration curves, and Decision Curve Analysis (DCA).

**Results:**

Six independent predictors were identified: ICU length of stay, minimum glucose, minimum sodium, maximum bicarbonate, minimum creatinine, and minimum diastolic blood pressure. The nomogram showed excellent discrimination, with AUCs of 0.926 (95% CI: 0.893–0.956), 0.922 (95% CI: 0.869–0.967), and 0.921 (95% CI: 0.895–0.944) in the training, internal validation, and external validation cohorts, respectively. Calibration analysis demonstrated good agreement, and DCA indicated clinical utility within a threshold probability range of 0–70%.

**Conclusions:**

This study developed a practical nomogram based on six readily available variables for dynamic in-hospital prediction of AKI in TBAD patients. The model may assist early risk stratification, although further prospective validation is warranted.

## Highlights

• A nomogram predicts acute kidney injury in type B aortic dissection.
• The model was developed in eICU-CRD and externally validated in MIMIC-IV.
• Six routinely available variables yielded AUCs above 0.92 across cohorts.
• Calibration and decision curve analyses supported clinical utility.

## Introduction

Aortic Dissection (AD) is a critical cardiovascular event characterized by the entry of blood through an intimal tear, leading to separation of the inner and medial layers of the vessel wall and the formation of true and false lumens.^1^^,^^2^ This condition is usually categorized according to the Stanford system as Type A Aortic Dissection (TAAD), which denotes involvement of the ascending aorta, and Type B Aortic Dissection (TBAD), which does not.^3^^,^^4^ AD frequently leads to Acute Kidney Injury (AKI), with reported perioperative incidence rates ranging from 15% to 25% for TAAD and 18% to 52.7% for TBAD.^5^^,^^6^ The development of AKI complicates clinical management in this patient population and leads to several serious adverse consequences: progression to chronic kidney disease; prolonged Intensive Care Unit (ICU) stay, and increased in-hospital mortality.^7^^,^^8^ These serious complications highlight the urgent need for early risk stratification and preventive strategies.

Although AKI is highly prevalent in TBAD, existing predictive models have predominantly focused on TAAD populations.^9^^,^^10^ Some studies have included mixed cohorts of both TAAD and TBAD patients for analysis.^11^ However, these approaches have largely overlooked the distinct pathophysiological mechanisms underlying AKI in these two conditions: AKI in TAAD is primarily related to surgical factors such as deep hypothermic circulatory arrest and cardiopulmonary bypass, whereas in TBAD it is more closely associated with non-surgical factors including refractory hypertension and renal artery involvement.^5^^,^^7^ Consequently, these models demonstrate limited generalizability for TBAD patients.

To address the issue, we leveraged detailed clinical data from two large-scale public databases ‒ the eICU Collaborative Research Database (eICU-CRD) and the Medical Information Mart for Intensive Care IV (MIMIC-IV) ‒ to identify independent predictors of AKI specifically within the TBAD population. We used these predictors to construct and validate an intuitive, clinically applicable nomogram for dynamic in-hospital individualized risk estimation.

## Materials and methods

### *Data source*

The cohort for training and internal validation was derived from eICU-CRD 2.0, whereas the cohort for external validation was obtained from the MIMIC-IV 3.1 database. The eICU-CRD includes over 200,000 ICU admissions from more than 200 U.S. hospitals (2014‒2015),^12^ while MIMIC-IV contains over 90,000 admissions from Beth Israel Deaconess Medical Center (2008‒2022).^13^ Author W.C. completed the required CITI program for ethical research conduct (Record ID: 67647740). This study utilized de-identified publicly available data and was therefore exempt from ethical approval. This observational cohort study is reported in accordance with the Strengthening the Reporting of Observational Studies in Epidemiology (STROBE) Statement.

### *Research population and data extraction*

Data were extracted using Navicat Premium (version 17.0.8; PremiumSoft CyberTech Ltd., Hong Kong) by executing PostgreSQL queries. Our study enrolled adult patients (aged 18 or above) diagnosed with aortic dissection according to International Classification of Diseases (ICD) codes (ICD-9: 441.01; ICD-10: I71.0, I71.1). Exclusion criteria consisted of: 1) A history of chronic kidney disease or AKI; 2) Incomplete clinical data (> 10% missing variables). For patients admitted multiple times, only the initial encounter was considered. The development of AKI during hospitalization was defined as the study endpoint.

The data collection encompassed the following categories: demographic characteristics (sex, age, height, weight, Body Mass Index [BMI]); hemodynamic parameters (Systolic Blood Pressure [SBP], Diastolic Blood Pressure [DBP]); laboratory indices (hemoglobin, white blood cell count, platelets, bicarbonate, glucose, calcium, creatinine, Blood Urea Nitrogen [BUN], anion gap, potassium, sodium, aspartate Aminotransferase [AST], International Normalized Ratio [INR], Alanine Aminotransferase [ALT]); and clinical outcomes (ICU length of stay, total hospital length of stay). For blood pressure and laboratory measurements, both peak and nadir values occurring between hospital admission and the first fulfillment of AKI diagnostic criteria were documented. For patients who developed AKI, only measurements obtained before AKI diagnosis were used as candidate predictors. Therefore, the present model should be interpreted as a dynamic in-hospital risk assessment tool rather than a strictly admission-only prediction model.

AKI was diagnosed according to established criteria^14^ as meeting any of the following: 1) Urine output below 0.5 mL/kg/h for 6 consecutive hours; 2) A 50% or greater increase in serum creatinine from baseline, occurring within 7-days; or 3) A rapid increment in serum creatinine of ≥ 26.5 µmoL/L (≥ 0.3 mg/dL) over a 48-hour period.

### *Development and validation of nomogram*

Our research constructed a predictive nomogram for AKI in TBAD population using the eICU-CRD database and conducted external validation with the MIMIC database. The eICU-CRD dataset was randomly assigned 70% of the training cohort and 30% of the internal validation cohort, with the MIMIC dataset utilized for external validation. Missing data were handled by excluding variables with > 10% missingness, with numerical variables imputed using median values and categorical variables using mode imputation. A three-stage variable selection strategy was implemented: initial feature screening via Least Absolute Shrinkage and Selection Operator (LASSO) regression, followed by univariate logistic regression (p < 0.1) to determine significant variables, and finally multivariate logistic regression (p < 0.05) to determine the final predictors. LASSO regression was used as an initial screening step to reduce redundancy among candidate variables and to mitigate the influence of multicollinearity. The selected predictors were used to build a nomogram. The model's performance was quantified across all datasets, with evaluation metrics including the Area Under the receiver operating characteristic Curve (AUC) for discrimination, calibration curves for agreement, and Decision Curve Analysis (DCA) for assessing clinical utility.

### *Statistical analysis*

R software (version 4.5.1; R Foundation for Statistical Computing, Vienna, Austria) was employed for all statistical computations in this study. Use the mean ± standard deviation to represent continuous data, and counts (percentages) to represent categorical data. For comparisons between groups, the Mann-Whitney *U*-test, Student's *t*-test, and Chi-Squared test were applied to non-normally distributed continuous, normally distributed continuous, and categorical variables, respectively. Confidence Intervals (CIs) for the AUC were calculated using the bootstrap method with 1000 replicates. Calibration was assessed through calibration plots. A two-tailed testing approach was adopted, and a p-value less than 0.05 was set as the statistical significance threshold.

## Results

### *Population characteristics*

The final analysis included 1,089 cases (training cohort: 387; internal validation cohort: 165, external validation cohort: 537). As shown in Table 1, there were significant differences (p < 0.05) in several baseline features among the training, internal validation, and external validation cohorts. Table 2 compares variable distributions between the AKI and non-AKI groups in the training set, with the following parameters exhibiting significant differences (p < 0.05): maximum and minimum hemoglobin, minimum platelet count, minimum white blood cell count, maximum bicarbonate, minimum anion gap, minimum BUN, minimum creatinine, minimum glucose, minimum sodium, maximum and minimum potassium, minimum INR, maximum and minimum SBP, maximum and minimum DBP, ICU length of stay, and total hospital length of stay.

**Table 1:** Variable distribution of training and validation sets.

**Variables**	**Overall** **(n = 1089)**	**Training Set** **(n = 387)**	**Internal Validation Set** **(n = 165)**	**External Validation Set** **(n = 537)**	**p**
Gender, n (%)					<0.001
Female	402 (36.9)	125 (32.3)	47 (28.5)	230 (42.8)	
Male	687 (63.1)	262 (67.7)	118 (71.5)	307 (57.2)	
Age (years)	65.49 ± 14.01	64.49 ± 14.19	65.14 ± 13.35	66.32 ± 14.05	0.139
Height (cm)	172.14 ± 11.14	173.20 ± 11.20	173.12 ± 10.61	170.64 ± 11.16	0.003
Weight (Kg)	72.19 ± 24.94	86.62 ± 23.19	87.47 ± 21.82	57.46 ± 17.10	<0.001
BMI (Kg/m^2)	25.59 ± 7.28	28.75 ± 7.00	29.15 ± 6.88	20.96 ± 4.78	<0.001
HB_max (g/dL)	14.66 ± 5.56	11.39 ± 1.87	11.65 ± 1.88	17.94 ± 6.16	<0.001
HB_min (g/dL)	9.46 ± 2.27	10.13 ± 2.05	10.28 ± 2.03	8.73 ± 2.27	<0.001
PLT_max (×10^9)	248.01 ± 137.42	183.18 ± 70.88	177.23 ± 72.78	316.48 ± 154.34	<0.001
PLT_min (×10^9)	141.15 ± 71.66	149.16 ± 63.65	139.50 ± 62.57	135.89 ± 78.94	0.02
WBC_max (×10^9)	14.24 ± 6.94	13.10 ± 5.22	13.65 ± 4.93	15.25 ± 8.29	<0.001
WBC_min (×10^9)	8.89 ± 4.41	9.99 ± 4.38	10.43 ± 4.26	7.63 ± 4.10	<0.001
BC_max (mmol/L)	27.05 ± 4.58	26.17 ± 3.74	25.98 ± 3.72	27.99 ± 5.13	<0.001
BC_min (mmol/L)	21.53 ± 4.10	22.70 ± 3.45	22.63 ± 3.70	20.37 ± 4.32	<0.001
AG_max (mmol/L)	14.01 ± 4.89	10.81 ± 3.54	10.93 ± 4.05	16.69 ± 4.11	<0.001
AG_min (mmol/L)	9.74 ± 3.48	9.09 ± 3.32	9.18 ± 3.94	10.26 ± 3.36	<0.001
Ca_max (mg/dL)	9.64 ± 2.88	9.97 ± 3.71	10.27 ± 4.12	9.19 ± 0.94	<0.001
Ca_min (mg/dL)	7.63 ± 1.34	7.29 ± 1.68	7.27 ± 1.60	8.01 ± 0.71	<0.001
BUN_max (mmol/L)	29.64 ± 24.63	20.92 ± 12.48	21.44 ± 13.36	38.44 ± 30.18	<0.001
BUN_min (mmol/L)	16.63 ± 10.27	16.50 ± 9.03	17.50 ± 10.86	16.46 ± 10.90	0.497
Creatinine_max (mg/dL)	1.70 ± 2.89	1.43 ± 3.51	1.42 ± 3.35	1.99 ± 2.13	0.006
Creatinine_min (mg/dL)	1.04 ± 0.81	1.02 ± 0.55	1.07 ± 0.70	1.04 ± 0.98	0.729
Glu_max (mg/dL)	177.18 ± 73.73	162.19 ± 56.25	166.65 ± 51.55	191.20 ± 86.86	<0.001
Glu_min (mg/dL)	101.33 ± 35.01	109.48 ± 34.35	108.86 ± 36.12	93.15 ± 33.26	<0.001
Na_max (mmol/L)	141.91 ± 5.16	140.73 ± 4.83	141.35 ± 4.68	142.94 ± 5.33	<0.001
Na_min (mmol/L)	135.84 ± 4.67	137.29 ± 4.55	137.81 ± 4.45	134.18 ± 4.23	<0.001
K_max (mmol/L)	4.72 ± 0.92	4.32 ± 0.64	4.32 ± 0.55	5.13 ± 1.01	<0.001
K_min (mmol/L)	3.63 ± 0.59	3.76 ± 0.50	3.77 ± 0.58	3.50 ± 0.62	<0.001
INR_max	10.25 ± 7.36	1.38 ± 0.64	1.39 ± 0.51	15.61 ± 3.24	<0.001
INR_min	1.18 ± 0.30	1.19 ± 0.25	1.19 ± 0.22	1.18 ± 0.32	0.884
ALT_max (U/L)	171.30 ± 457.69	98.84 ± 409.81	68.58 ± 93.78	223.15 ± 509.01	0.003
ALT_min (U/L)	25.27 ± 34.54	26.99 ± 36.14	38.85 ± 71.07	21.97 ± 19.92	0.001
AST_max (U/L)	186.91 ± 602.66	98.84 ± 409.81	68.58 ± 93.78	248.72 ± 712.94	0.008
AST_min (U/L)	36.70 ± 115.16	26.99 ± 36.14	38.85 ± 71.07	40.70 ± 142.04	0.456
SBP_max (mmHg)	164.00 ± 34.90	167.68 ± 37.77	167.67 ± 42.88	160.22 ± 29.23	0.002
SBP_min (mmHg)	88.37 ± 21.34	92.76 ± 24.05	89.88 ± 21.37	84.73 ± 18.44	<0.001
DBP_max (mmHg)	95.97 ± 29.38	95.83 ± 34.51	90.58 ± 31.90	97.73 ± 23.91	0.024
DBP_min (mmHg)	44.32 ± 11.66	46.55 ± 11.64	45.30 ± 11.10	42.41 ± 11.55	<0.001
ICU_los_days (days)	5.35 ± 7.09	3.84 ± 4.65	3.67 ± 4.04	6.95 ± 8.75	<0.001
Hospital_los (days)	9.83 ± 10.44	7.23 ± 6.15	7.65 ± 6.07	12.37 ± 13.04	<0.001

BMI, Body Mass Index; SBP, Systolic Blood Pressure; DBP, Diastolic Blood Pressure; PLT, Platelets; WBC, White Blood Cell; HB, Hemoglobin; BC, Bicarbonate; AG, Anion Gap; Ca, Calcium; Glu, Glucose; BUN, Blood Urea Nitrogen; K, Potassium; Na, Sodium; AST, Aspartate Aminotransferase; INR, International Normalized Ratio; ALT, Alanine Aminotransferase; ICU_los_days, ICU length of stay; Hospital_los, total Hospital length of stay.

**Table 2:** Differences in variable distribution between AKI and non AKI groups in the training set.

**Variables**	**Non-AKI group** **(n = 295)**	**AKI group** **(n = 92)**	**p**
Gender, n(%)			0.649
Female	93 (31.5)	32 (34.8)	
Male	202 (68.5)	60 (65.2)	
Age (years)	65.20 ± 13.88	62.21 ± 14.99	0.077
Height (cm)	173.44 ± 11.08	172.42 ± 11.60	0.443
Weight (Kg)	86.25 ± 22.16	87.83 ± 26.34	0.574
BMI (Kg/m^2)	28.55 ± 6.56	29.40 ± 8.26	0.318
HB_max (g/dL)	11.55 ± 1.74	10.87 ± 2.17	0.002
HB_min (g/dL)	10.02 ± 2.01	10.50 ± 2.14	0.049
PLT_max (×10^9)	185.66 ± 68.56	175.22 ± 77.69	0.218
PLT_min (×10^9)	142.36 ± 56.77	170.96 ± 78.38	<0.001
WBC_max (×10^9)	13.20 ± 5.03	12.75 ± 5.81	0.473
WBC_min (×10^9)	9.27 ± 3.63	12.33 ± 5.62	<0.001
BC_max (mmol/L)	26.80 ± 3.66	24.20 ± 3.26	<0.001
BC_min (mmol/L)	22.64 ± 3.36	22.88 ± 3.74	0.571
AG_max (mmol/L)	10.71 ± 3.47	11.12 ± 3.75	0.379
AG_min (mmol/L)	8.45 ± 2.90	11.11 ± 3.77	<0.001
Ca_max (mg/dL)	10.02 ± 3.76	9.82 ± 3.55	0.648
Ca_min (mg/dL)	7.21 ± 1.69	7.53 ± 1.63	0.116
BUN_max (mmol/L)	21.32 ± 12.73	19.62 ± 11.59	0.254
BUN_min (mmol/L)	15.54 ± 7.85	19.60 ± 11.59	<0.001
Creatinine_max (mg/dL)	1.48 ± 4.00	1.27 ± 0.65	0.611
Creatinine_min (mg/dL)	0.94 ± 0.49	1.27 ± 0.65	<0.001
Glu_max (mg/dL)	164.89 ± 53.31	153.52 ± 64.34	0.091
Glu_min (mg/dL)	102.66 ± 25.93	131.35 ± 46.90	<0.001
Na_max (mmol/L)	140.59 ± 4.77	141.15 ± 5.02	0.333
Na_min (mmol/L)	136.30 ± 3.88	140.46 ± 5.07	<0.001
K_max (mmol/L)	4.38 ± 0.62	4.13 ± 0.66	0.001
K_min (mmol/L)	3.69 ± 0.44	3.97 ± 0.59	<0.001
INR_max	1.41 ± 0.71	1.31 ± 0.36	0.321
INR_min	1.15 ± 0.19	1.29 ± 0.35	<0.001
ALT_max (U/L)	111.22 ± 465.05	56.56 ± 43.55	0.483
ALT_min (U/L)	25.05 ± 37.46	33.64 ± 30.75	0.211
AST_max (U/L)	111.22 ± 465.05	56.56 ± 43.55	0.483
AST_min (U/L)	25.05 ± 37.46	33.64 ± 30.75	0.211
SBP_max (mmHg)	174.94 ± 36.77	144.40 ± 31.09	<0.001
SBP_min (mmHg)	89.11 ± 21.64	104.48 ± 27.56	<0.001
DBP_max (mmHg)	102.52 ± 34.88	74.39 ± 22.60	<0.001
DBP_min (mmHg)	44.53 ± 10.50	53.05 ± 12.75	<0.001
ICU_los_days (days)	2.98 ± 3.15	6.62 ± 7.02	<0.001
Hospital_los (days)	5.96 ± 4.51	11.32 ± 8.50	<0.001

AKI, Acute Kidney Injury; BMI, Body Mass Index; SBP, Systolic Blood Pressure; DBP, Diastolic Blood Pressure; WBC, White Blood Cell; PLT, Platelets; HB, Hemoglobin; BC, Bicarbonate; AG, Anion Gap; Ca, Calcium; Glu, Glucose; BUN, Blood Urea Nitrogen; K, Potassium; Na, Sodium; AST, Aspartate Aminotransferase; INR, International Normalized Ratio; ALT, Alanine Aminotransferase; ICU_los_days, ICU length of stay; Hospital_los, total Hospital length of stay.

### *Risk factor analysis*

Initially, a 5-fold cross-validated LASSO regression was applied to the training set variables (Fig. 1A and 1B), which identified 29 variables with non-zero coefficients. These variables were subsequently subjected to univariate analysis, as summarized in Table 3. Variables yielding a p-value < 0.1 were chosen for further multivariate analysis. Ultimately, six independent predictors were retained (Table 4) and incorporated into the construction of the nomogram.

**Fig. 1:**
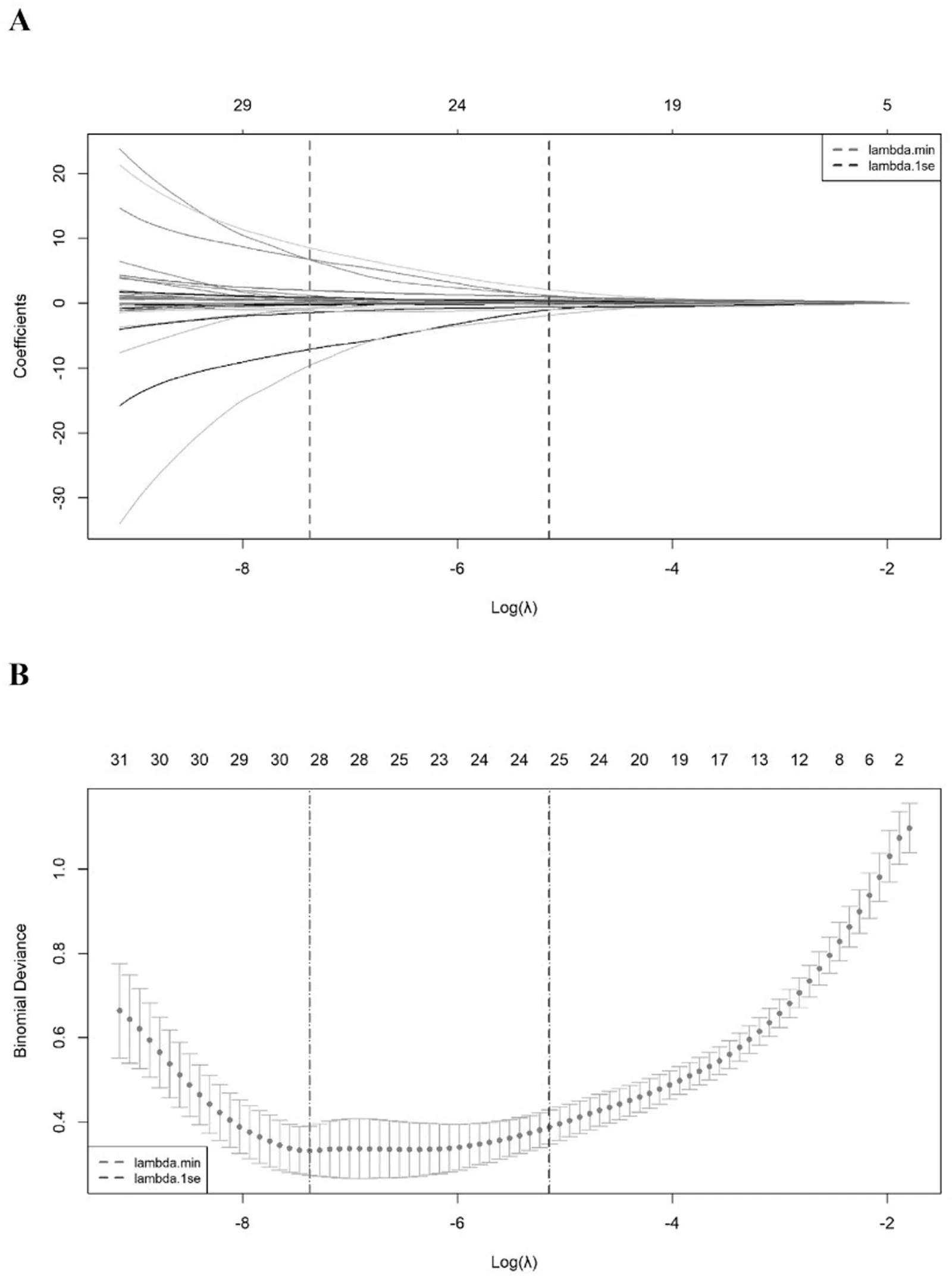
**Predictor selection using least absolute shrinkage and selection operator (LASSO) regression.** (A) LASSO coefficient profiles of the candidate predictors across different values of the regularization parameter λ. The horizontal axis represents log(λ), and the vertical axis represents the standardized regression coefficients. (B) Selection of the optimal regularization parameter using five-fold cross-validation. The horizontal axis represents log(λ), the vertical axis represents the binomial deviance, and the numbers along the upper axis indicate the number of non-zero coefficients. The red and blue dashed lines indicate lambda.min, which minimizes the cross-validation error, and lambda.1se, which represents the most parsimonious model within one standard error of the minimum, respectively. Lambda.min was selected for subsequent predictor screening. LASSO, Least Absolute Shrinkage and Selection Operator.

**Table 3:** The results of univariate analysis.

**Variables**	**Coefficient**	**Std_Error**	***z*_Value**	**p**	**OR**	**95% CI**
Height	-0.0919	0.1195	-0.769	0.442	0.912	(0.721‒1.153)
Weight	0.0658	0.1181	0.557	0.577	1.068	(0.844‒1.344)
BMI	0.1152	0.1163	0.99	0.322	1.122	(0.89‒1.407)
HB_max	-0.3846	0.1268	-3.032	0.002	0.681	(0.528‒0.869)
HB_min	0.233	0.1189	1.959	0.05	1.262	(1‒1.597)
PLT_max	-0.1548	0.1256	-1.233	0.218	0.857	(0.664‒1.089)
PLT_min	0.4238	0.1163	3.643	<0.001	1.528	(1.219‒1.927)
WBC_max	-0.0886	0.1232	-0.719	0.472	0.915	(0.713‒1.158)
WBC_min	0.6935	0.1313	5.281	<0.001	2.001	(1.56‒2.614)
BC_max	-0.8993	0.1597	-5.633	<0.001	0.407	(0.294‒0.55)
BC_min	0.0693	0.1212	0.572	0.567	1.072	(0.848‒1.366)
Ca_max	-0.0592	0.1238	-0.478	0.633	0.943	(0.729‒1.189)
Ca_min	0.199	0.1257	1.584	0.113	1.22	(0.959‒1.573)
BUN_min	0.412	0.1215	3.392	<0.001	1.51	(1.201‒1.934)
Creatinine_max	-0.0787	0.1591	-0.495	0.621	0.924	(0.543‒1.173)
Creatinine_min	0.6615	0.1558	4.246	<0.001	1.938	(1.453‒2.67)
Glu_max	-0.2409	0.1428	-1.686	0.092	0.786	(0.584‒1.021)
Glu_min	0.9011	0.1475	6.111	<0.001	2.462	(1.865‒3.33)
Na_max	0.1135	0.1172	0.969	0.333	1.12	(0.888‒1.408)
Na_min	1.0988	0.1618	6.79	<0.001	3	(2.218‒4.19)
K_min	0.6126	0.1328	4.614	<0.001	1.845	(1.432‒2.414)
SBP_max	-1.1761	0.1842	-6.386	<0.001	0.308	(0.211‒0.435)
SBP_min	0.6321	0.127	4.979	<0.001	1.882	(1.477‒2.434)
DBP_max	-1.604	0.2584	-6.207	<0.001	0.201	(0.117‒0.323)
DBP_min	0.747	0.1313	5.688	<0.001	2.111	(1.644‒2.756)
ICU_los_days	0.7651	0.1433	5.337	<0.001	2.149	(1.648‒2.895)
Hospital_los	0.8945	0.1454	6.152	<0.001	2.446	(1.862‒3.294)
Gender Female	0.1471	0.2522	0.583	0.56	1.158	(0.701‒1.89)
Gender Male	-0.1471	0.2522	-0.583	0.56	0.863	(0.529‒1.426)

Std_Error, Standard Error; OR, Odds Ratio; CI, Confidence Interval; BMI, Body Mass Index; PLT, Platelets; HB, Hemoglobin; BC, Bicarbonate; WBC, White Blood Cell; Ca, Calcium; BUN, Blood Urea Nitrogen; Glu, Glucose; K, Potassium; Na, Sodium; SBP, Systolic Blood Pressure; DBP, Diastolic Blood Pressure; ICU_los_days, ICU length of stay; Hospital_los, total Hospital length of stay.

**Table 4:** The results of multivariate analysis.

**Variables**	**Coefficient**	**Std_Error**	***z*_Value**	**p**	**OR**	**95%CI**
ICU_los_days	1.4287	0.2608	5.479	<0.001	4.173	(2.61‒7.278)
Glu_min	0.7819	0.1906	4.103	<0.001	2.186	(1.521‒3.223)
Na_min	1.2272	0.2242	5.474	<0.001	3.412	(2.257‒5.453)
BC_max	-1.1421	0.2481	-4.602	<0.001	0.319	(0.19‒0.505)
Creatinine_min	0.4974	0.1431	3.475	<0.001	1.644	(1.239‒2.236)
DBP_min	0.9805	0.1926	5.091	<0.001	2.666	(1.852‒3.959)

Std_Error, Standard Error; OR, Odds Ratio; CI, Confidence Interval; BC, Bicarbonate; Glu, Glucose; Na, Sodium; DBP, Diastolic Blood Pressure; ICU_los_days, ICU length of stay.

### *Construction and evaluation of nomogram*

The developed nomogram is presented in Fig. 2. Each predictor incorporated into the model is assigned a specific point value on the nomogram scale. By aggregating the point values from all predictors, the overall score can be converted into an estimated probability of AKI occurrence.

**Fig. 2:**
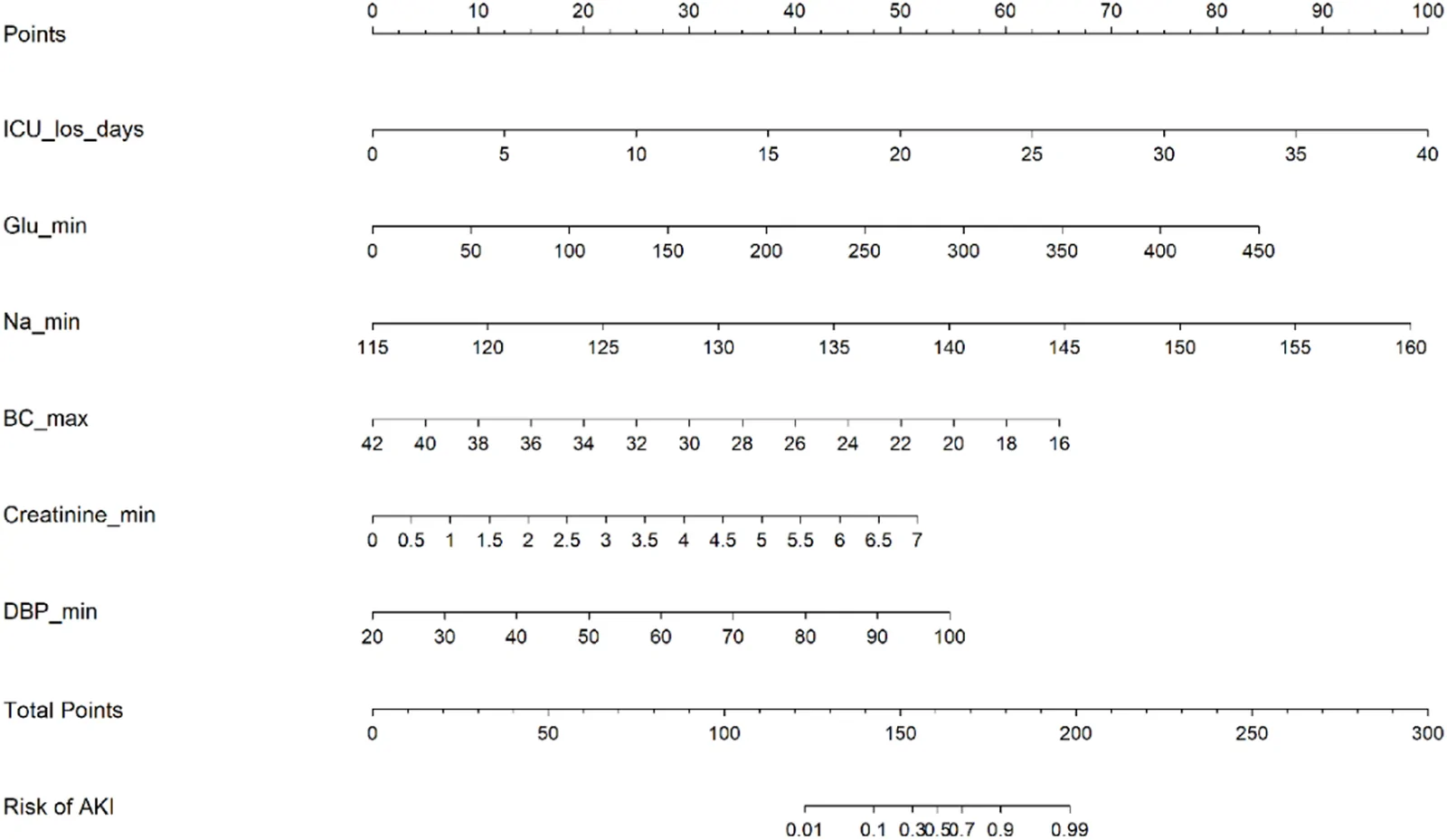
Predictive nomogram model. The total scores of all variables were matched on the nomogram to obtain the probability of AKI occurrence. BC, Bicarbonate; Glu, Glucose; Na, Sodium; DBP, Diastolic Blood Pressure; ICU_los_days, ICU length of stay; AKI, Acute Kidney Injury.

As shown in Fig. 3, the nomogram demonstrates strong discriminative ability, manifested as a good AUC (training: 0.926 (95% CI: 0.893‒0.956); internal validation: 0.922 (95% CI: 0.869‒0.967); external validation: 0.921 (95% CI: 0.895‒0.944). Detailed metrics are provided in Table 5. The calibration curves for all cohorts corresponded well with the reference diagonal (Fig. 4), suggesting high accuracy in probability estimation. Moreover, DCA (Fig. 5) confirmed the model's clinical value by demonstrating a net benefit across a threshold probability range of 0–70%.

**Fig. 3:**
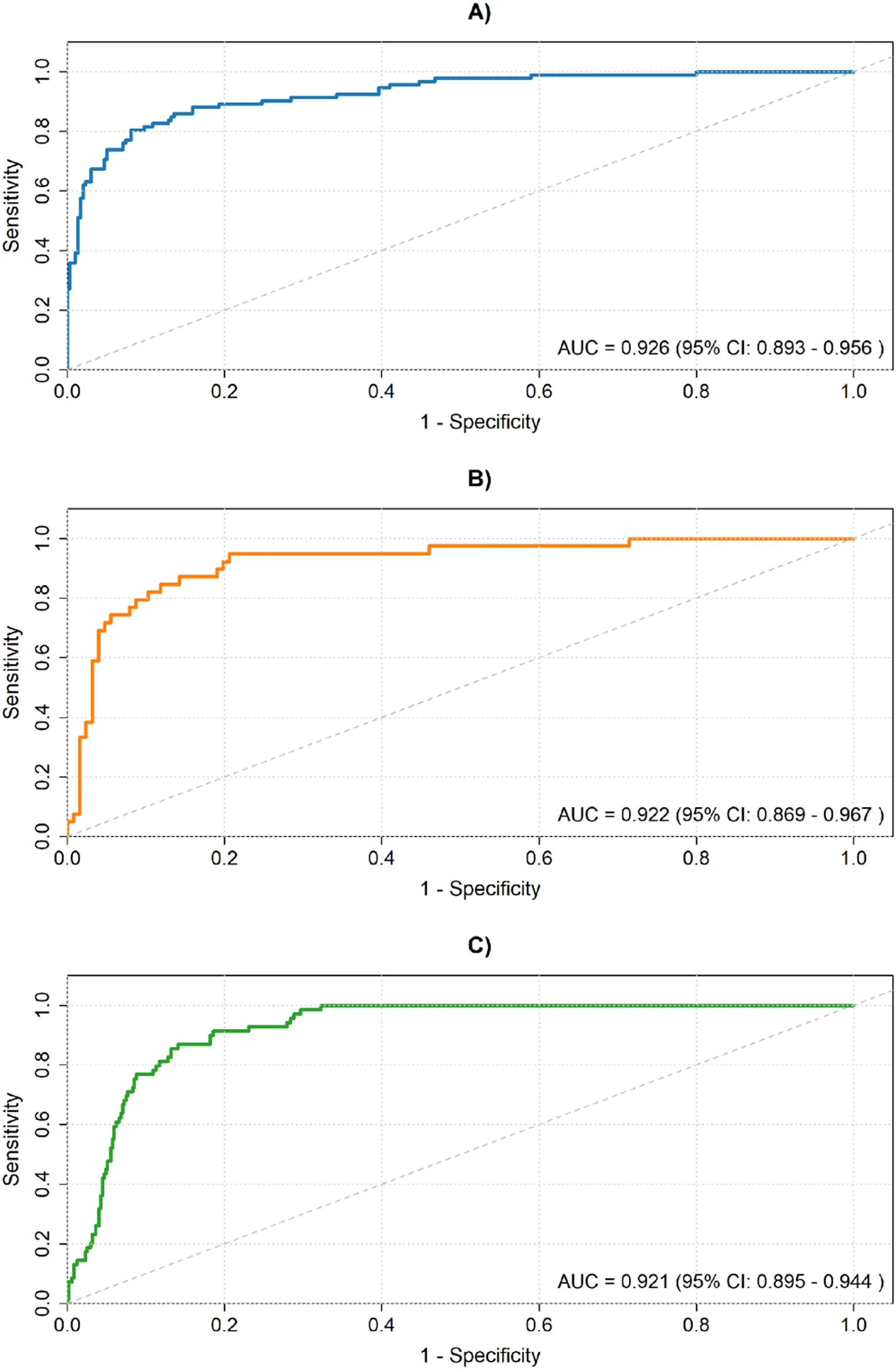
ROC curves of nomogram. The discriminative ability of the model can be evaluated through AUC. (A) Training cohort; (B) Internal validation cohort; (C) External validation cohort. ROC, Receiver Operating Characteristic; AUC, The Area Under the receiver operating characteristic Curve.

**Table 5:** Performance of the nomogram.

**Dataset**	**Accuracy**	**Precision**	**Recall**	**F1_Score**	**AUC**	**95%CI**
Training Set	0.894	0.849	0.674	0.752	0.926	(0.894‒0.958)
Internal Validation Set	0.891	0.818	0.692	0.75	0.922	(0.873‒0.972)
External Validation Set	0.886	0.71	0.657	0.682	0.921	(0.896‒0.946)

CI, Confidence Interval; AUC, The Area Under the receiver operating characteristic Curve.

**Fig. 4:**
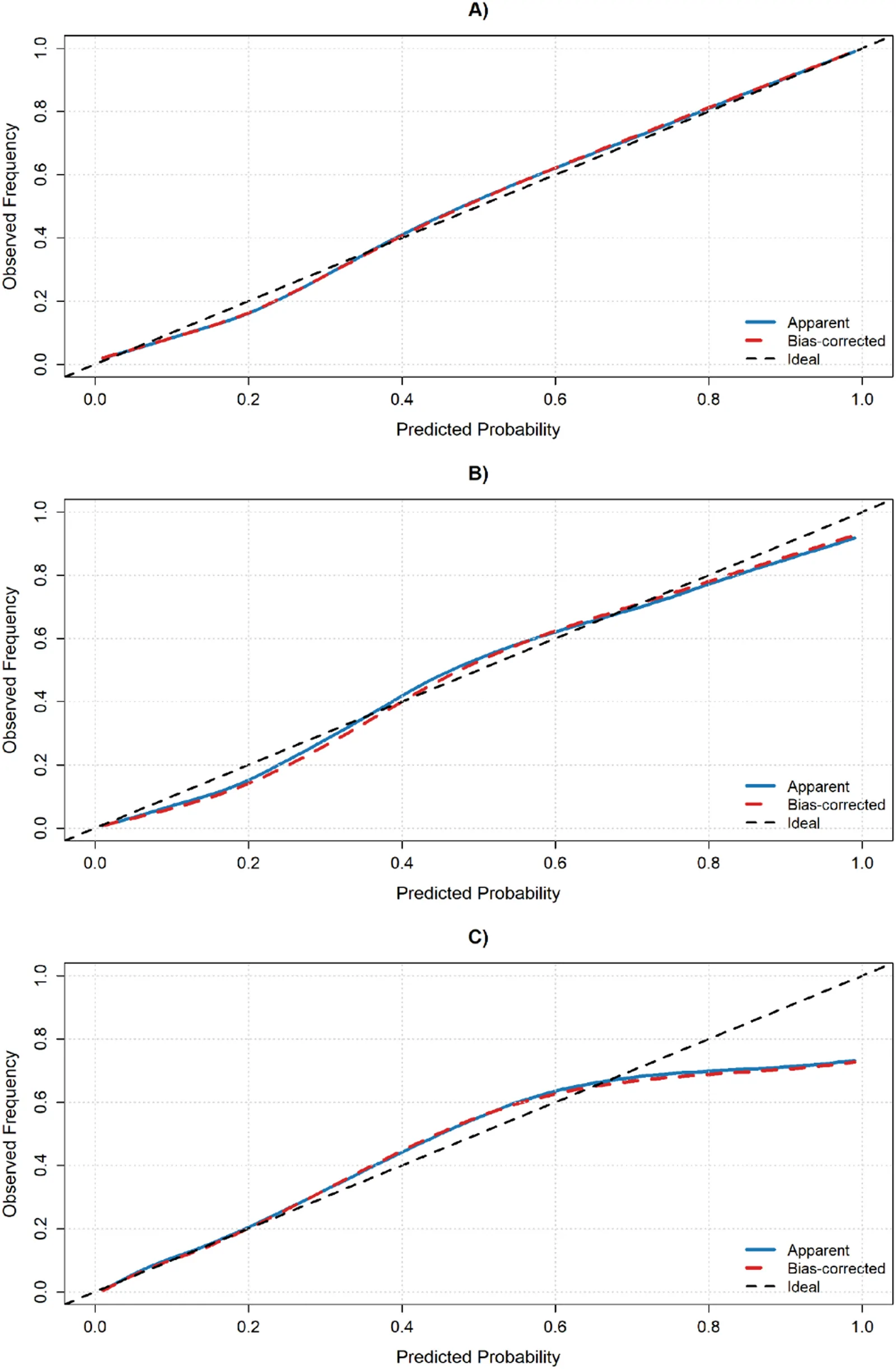
Calibration curves of nomogram. The calibration performance of the AKI prediction model was assessed by examining the agreement between the predicted probabilities and the actual observed frequencies. (A) Training cohort; (B) Internal validation cohort; (C) External validation cohort. AKI, Acute Kidney Injury.

**Fig. 5:**
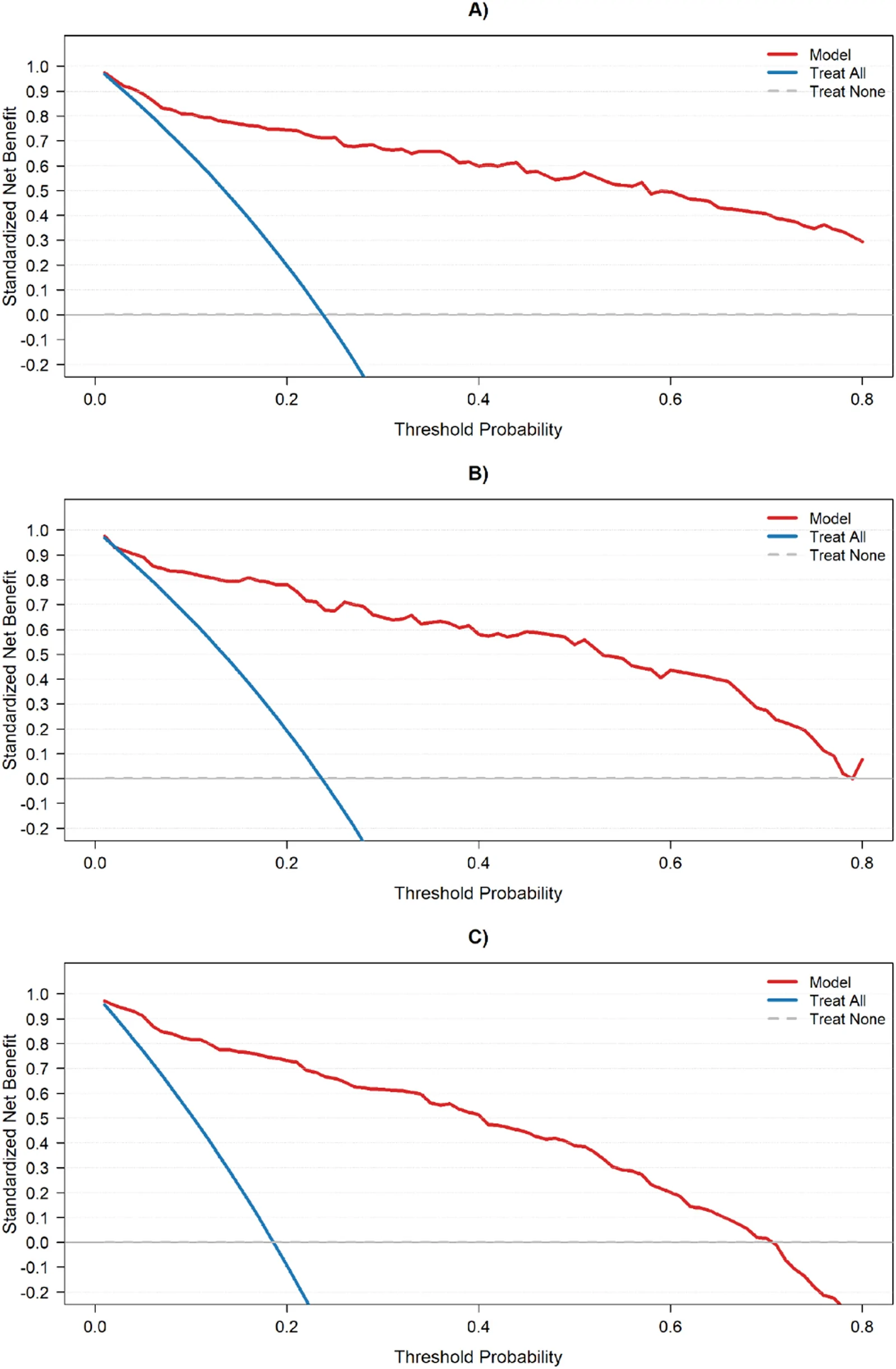
DCA curves of nomogram. The vertical axis indicates net profit, and the horizontal axis indicates threshold probability. (A) Training cohort; (B) Internal validation cohort; (C) External validation cohort. DCA, Decision Curve Analysis.

## Discussion

Based on two large-scale publicly available databases, our research constructed and extensively validated a parsimonious nomogram for AKI prediction specifically in TBAD patients. The model maintained high discriminative ability across both internal and external validation and demonstrated substantial clinical utility. By incorporating routinely available clinical parameters, it offers a practical solution for early AKI risk assessment in this patient population.

Our findings identified ICU length of stay as the strongest variable associated with AKI in TBAD patients (OR = 4.173, 95% CI: 2.61–7.278, p < 0.001), indicating that each additional day in the ICU was associated with an approximately fourfold increase in AKI risk. Prolonged ICU stay not only reflects the need for ongoing organ support and monitoring but may also reflect greater disease severity or a more complicated in-hospital course and extends exposure to potential renal insults, such as fluid overload and nephrotoxic medications.^15^ Although multivariable analysis was performed to mitigate confounding, the interpretation of this association should still consider the possibility of reverse causality ‒ where AKI itself may lead to longer ICU stays.^16^ Therefore, ICU length of stay in the present model should be interpreted cautiously and may be better understood as a dynamic in-hospital risk-associated marker rather than a purely upstream causal predictor. Regardless, these results suggest that TBAD patients with an anticipated prolonged ICU stay warrant heightened vigilance for AKI, prompting earlier implementation of intensive renal monitoring and preventive strategies.

Extensive evidence indicates that hyperglycemia contributes to the development of AKI.^17^^,^^18^ Consistent with these findings, our results indicate that a higher minimum glucose value is linked to a heightened risk of AKI (OR = 2.186, 95% CI: 1.521–3.223, p < 0.001). The detrimental effects of hyperglycemia on renal function may be mediated through several pathways. First, hyperglycemia impairs endothelial function and increases vascular permeability, thereby reducing renal perfusion.^19^ Second, it induces oxidative stress and exacerbates inflammatory responses, further compromising kidney blood flow.^20^^,^^21^ Finally, hyperglycemia is closely linked to atherosclerosis, which may promote renal artery stenosis and further diminish renal perfusion.^22^ These findings emphasize the importance of actively controlling blood sugar levels in TBAD patients to reduce the risk of AKI.

Existing evidence supports a correlation between increased serum sodium levels and the occurrence of AKI. The underlying mechanisms primarily involve sodium-induced vasoconstriction and intravascular dehydration, both of which can contribute to renal dysfunction.^23^^,^^24^ Clinical observations further support a positive correlation between hypernatremia and increased AKI risk. As demonstrated by Kumar et al.,^25^ patients with subarachnoid hemorrhage showed progressively higher AKI risk with each incremental 1 mmol/L rise in serum sodium levels, specifically a 5.4% increase per unit. Similarly, Wang et al.^26^ identified hypernatremia as an independent risk factor for AKI in acute myocardial infarction patients. Our study yielded consistent findings in the TBAD population, demonstrating that higher minimum sodium levels were associated with a higher risk of AKI (OR = 3.412, 95% CI: 2.257–5.453, p < 0.001). These results reinforce the association between serum sodium and AKI risk and highlight the importance of closely monitoring sodium dynamics during the perioperative management of TBAD patients.

An increased maximum bicarbonate concentration was identified as an independent variable inversely associated with AKI in the TBAD population (OR = 0.319, 95% CI: 0.19–0.505, p < 0.001). This finding aligns with earlier research: Jung et al.^27^ reported that preoperative bicarbonate levels below 23 mEq/L were independently associated with AKI and prolonged ICU stay in cardiac surgery patients. Similarly, prospective research by Ribeiro et al.^28^ involving critically ill patients indicated that low bicarbonate levels (< 22 mEq/L) not only increased AKI risk but were also significantly linked to poor renal recovery. Notably, although pathophysiological mechanisms suggest a potential protective effect of higher bicarbonate levels, a systematic review by Tie et al.^29^ demonstrated that perioperative prophylactic sodium bicarbonate infusion failed to reduce the incidence of cardiac surgery-associated AKI and might even prolong mechanical ventilation and ICU stay. This implies that merely correcting serum bicarbonate may be insufficient to improve renal outcomes in complex clinical settings. In summary, our study suggests that higher bicarbonate levels were inversely associated with AKI risk in TBAD patients and underscores the importance of monitoring its dynamic changes in clinical management. However, whether it can serve as an effective target for therapeutic intervention warrants further investigation.

Creatinine, a fundamental and traditional marker of renal function, has demonstrated significant value in AKI risk assessment in various clinical settings. Yue et al.^30^ confirmed, through a machine learning algorithm, that minimum creatinine level is a key predictor in models for sepsis-associated AKI. Similarly, Luo et al.^9^ incorporated preoperative creatinine as a core variable in a prediction model for severe AKI following TAAD surgery. Our results corroborate these earlier observations, with the minimum creatinine level emerging as a significant variable associated with AKI among TBAD patients (OR = 1.644, 95% CI: 1.239–2.236, p < 0.001). However, this finding should be interpreted cautiously, as creatinine-related variables may also reflect baseline renal vulnerability or early renal functional changes in this population. These consistent results underscore the clinical importance of timely identifying patients with abnormal creatinine-related changes. Implementing proactive renal protective strategies in this high-risk group may help reduce the incidence of AKI.

Our study indicated that higher minimum DBP (OR = 2.666, 95% CI: 1.852‒3.959; p < 0.001) is associated with AKI in patients with TBAD. While previous studies have predominantly focused on SBP,^31^^,^^32^ our findings suggest that diastolic pressure may also be relevant to AKI risk independently of systolic blood pressure. However, the underlying mechanism remains uncertain, and this association should be interpreted cautiously. It may reflect hemodynamic burden, disease severity, or residual confounding rather than a direct causal effect. Consequently, in the management of TBAD patients, along with strict systolic pressure control, diastolic pressure may also merit close monitoring. Further studies are needed to determine whether dual-target blood pressure management could reduce AKI risk in clinical practice.

This study has several limitations. First, as a retrospective study based on public ICU databases, it is subject to residual confounding and potential selection bias. Second, although all candidate predictors were extracted before AKI diagnosis, some dynamic variables may still represent early changes during the evolution of AKI rather than purely antecedent risk factors. In addition, the exact interval from ICU admission to AKI onset was not separately quantified in the present study, which limited a more detailed assessment of temporal relationships. Third, several clinically relevant variables, such as treatment strategy, dissection extent, malperfusion status, and detailed comorbidity information, were not consistently available in the source databases. Fourth, admission creatinine or baseline estimated glomerular filtration rate was not explicitly modeled, which may have limited adjustment for baseline renal vulnerability. Finally, because both cohorts were derived from U.S.-based ICU populations, the generalizability of the present findings to other ethnic, socioeconomic, and healthcare settings remains to be further established.

## Conclusions

In this research, we have constructed and rigorously validated a novel nomogram that predicts AKI risk specifically in TBAD patients. Utilizing six readily available clinical variables, this practical tool demonstrated reliable performance in the validation cohorts. It may assist dynamic in-hospital risk stratification, although prospective validation and broader external validation are required before routine clinical implementation.

## Ethics approval and consent to participate

Not applicable.

## Author’s contribution

Weijian Chen: Conceptualization; data curation; formal analysis; methodology; software; visualization; writing-original draft.

Zhengfei Li: Investigation; validation; writing-review & editing.

Xiaojun Shu: Conceptualization; funding acquisition; project administration; supervision; writing-review & editing.

All authors read and approved the final manuscript.

## Funding

This research was funded by Science and Technology Plan Project of Gansu Province (21JR7RA358); and the fund of the First Hospital of Lanzhou University (ldyyyn2021-64).

## Declaration of competing interest

The authors declare no conflicts of interest.

## Data Availability

The data that support the findings of this study are available from the corresponding author upon reasonable request.
